# Comprehensive evaluation of preventive medicine talent in Chinese medical vocational colleges based on the entropy weight method

**DOI:** 10.3389/fmed.2026.1749127

**Published:** 2026-05-20

**Authors:** Tuo Qiu, Hong Li, Xinmei Yang, Qi Lin, Dan Li, Aizhen Chen, Mingjun Chen, Hailin Zhuang

**Affiliations:** School of Public Health and Health Management, Fujian Health College, Fuzhou, Fujian, China

**Keywords:** entropy weight method, medical vocational education, preventive medicine talent, regional education development, talent evaluation

## Abstract

**Objectives:**

With the increase in the mortality rate associated with noncommunicable diseases (NCDs), the development of preventive medicine talent has become a top priority. In this study, the quality of preventive medicine talent was evaluated on the basis of a vocational education program that was launched by the Chinese government in 2023.

**Methods:**

A cross-sectional survey consisting of self-designed questionnaires was administered for this study, and 20 medical colleges and 1,083 graduate students were included. The questionnaire consisted of four sections: personal qualities (PQs), professional knowledge (PK), professional skills (PSs), and comprehensive competencies (CCs). We used the entropy weight method to objectively assess the quality of the preventive medicine talent.

**Results:**

The average score for talent quality in China was 88.39 points. The highest-ranked school was located in Shandong Province, with a score surpassing 95 points in our evaluation system for talent quality. Talent quality was highest in central China (89.4), followed by eastern China (89.11) and western China (85.16). The average PQ, PK, PS, and CC scores were 12.64, 26.93, 35.79 and 12.64 points, respectively, with PQ scores exhibiting the highest standard deviation (SD = 2.05).

**Conclusion:**

The national talent quality score was deemed acceptable, with the top-performing college located in Shandong. Regional disparities were evident, with higher scores in eastern/central China than in western China, which is potentially attributable to regional development gaps. Recommendations for improving talent quality include optimizing curricula for workplace relevance, strengthening public health and preventive medicine accreditation and expanding the prescribing authority of public health physicians.

## 1 Introduction

With changes in the Chinese chronic disease spectrum and the development of population aging, the national disease burden is changing from early death to long-term illness. From 1990 to 2017, the number of cases involving infectious, maternal, neonatal, and nutritional diseases decreased by 1.2 million. However, the number of deaths due to noncommunicable diseases (NCDs) significantly increased from 5.9 million in 1990 to 7.9 million in 2017. NCDs have been the main cause of death in China since the 1990s (1).

Preventive medicine, which focuses on preventing the occurrence of disease, can effectively address these challenges. Talent quality constitutes the external performance of the knowledge, capabilities and literacy of preventive medicine talent. Sufficient talent quality is required to promote public health practices and to improve the health of the population. Therefore, China has set higher requirements for talent quality in the new era. According to the guidance of the Medium- and Long-term Plan for the Development of Medical and Health Talent (2011–2020) issued by the Chinese Ministry of Health in 2011, the number of persons in professional public health institutions per thousand persons should reach 0.83 by 2020 (2). However, according to the 2023 China Health Yearbook, this number was only 0.69 by 2022, which means that the requirement has not been met (3).

Scholars have researched the capabilities of public health talent. In 2005, the Standards Development Committee proposed a mature evaluation system for public health talent, which included job competencies, such as theoretical knowledge, communication, and leadership (4). The Spanish Association of Public Health and Healthcare assesses health needs, formulates health policies, and ensures the quality of health care services. On this basis, the association sets requirements for the comprehensive knowledge, abilities, and qualities of public health talent, which are used to guide education and training (5). In a quantitative survey, Leethongdissakul et al. used exploratory factor analysis to test the competencies of public health professionals, including five main proficiencies: (1) public health administration and laws; (2) disease prevention and control; (3) social and environmental determinants of health and health research; (4) health promotion and the community; and (5) basic medical care, screening, and diagnosis (6). Nelson-Hurwitz et al. conducted research on undergraduate core public health courses. Through the development and testing of a series of three introductory public health courses, they assessed students’ mastery of the required concepts and skills and then adjusted the courses on the basis of student feedback (7). Brînzac et al. conducted an online survey targeting European public health students and early-career professionals, with 127 participants from 25 countries included in the analysis. Health promotion, science and practice, and leadership combined with systems thinking emerged as the top-ranked critical competency domains for future career development (8). Warren et al. used a modified Delphi technique to identify public health competencies for Australian pharmacists (9). Yunfeng et al. surveyed a public health talent cultivation system based on competencies (10). Li et al. established a curriculum system for undergraduate students to develop innovative practical skills in public health; this system plays a substantial role in improving the knowledge structure of public health professionals and meeting employment needs (11).

Some scholars concentrate on a specific public health capability. Torok et al. surveyed competencies for detecting, investigating, and responding to foodborne illness outbreaks (12). Hughes focused on competencies for effective public health nutrition practices (13).

Some scholars have focused on evaluating the level of training capabilities. Van der Putten et al. developed a questionnaire to analyze the necessary levels of skill mastery in terms of core public health competencies among various personnel types in Thailand. The findings indicated significant variations in skill mastery expectations across different staff levels (14). Adewale et al. conducted an online self-administered survey and reported that public health employees reported relatively high proficiency in foundational public health informatics (15).

These articles concentrated on the development of competency statements or frameworks for public health and training public health students or practitioners to develop competencies. However, a comprehensive evaluation of these studies is lacking. In our study, we comprehensively evaluated preventive medicine talent in China on the basis of the entropy weight method. On the basis of the uncertainty contained within each variable, we determined the weight of each indicator, thereby eliminating subjective interference and obtaining an objective result.

## 2 Materials and methods

### 2.1 Study setting and design

Preventive medicine is a national-controlled medical major (marked with “K”) in Chinese medical vocational colleges, and the establishment of a program requires strict approval from the Ministry of Education (MOE) (16). The Preventive Medicine program resumed enrolling students at the higher vocational level in 2016. In accordance with the official results from the Ministry of Education regarding the approval of programs at vocational colleges, the number of vocational colleges newly approved to offer this program in 2016, 2017, 2018, and 2019 was 4, 6, 7, and 7, respectively (17–20). By 2019, a total of 25 medical vocational colleges in China had received approval to offer a degree program in preventive medicine (21).

A cross-sectional analysis method was used to survey graduate preventive medicine students in China from May to September 2023. We used the multistage cluster sampling method to recruit students.

**Figure 1:**
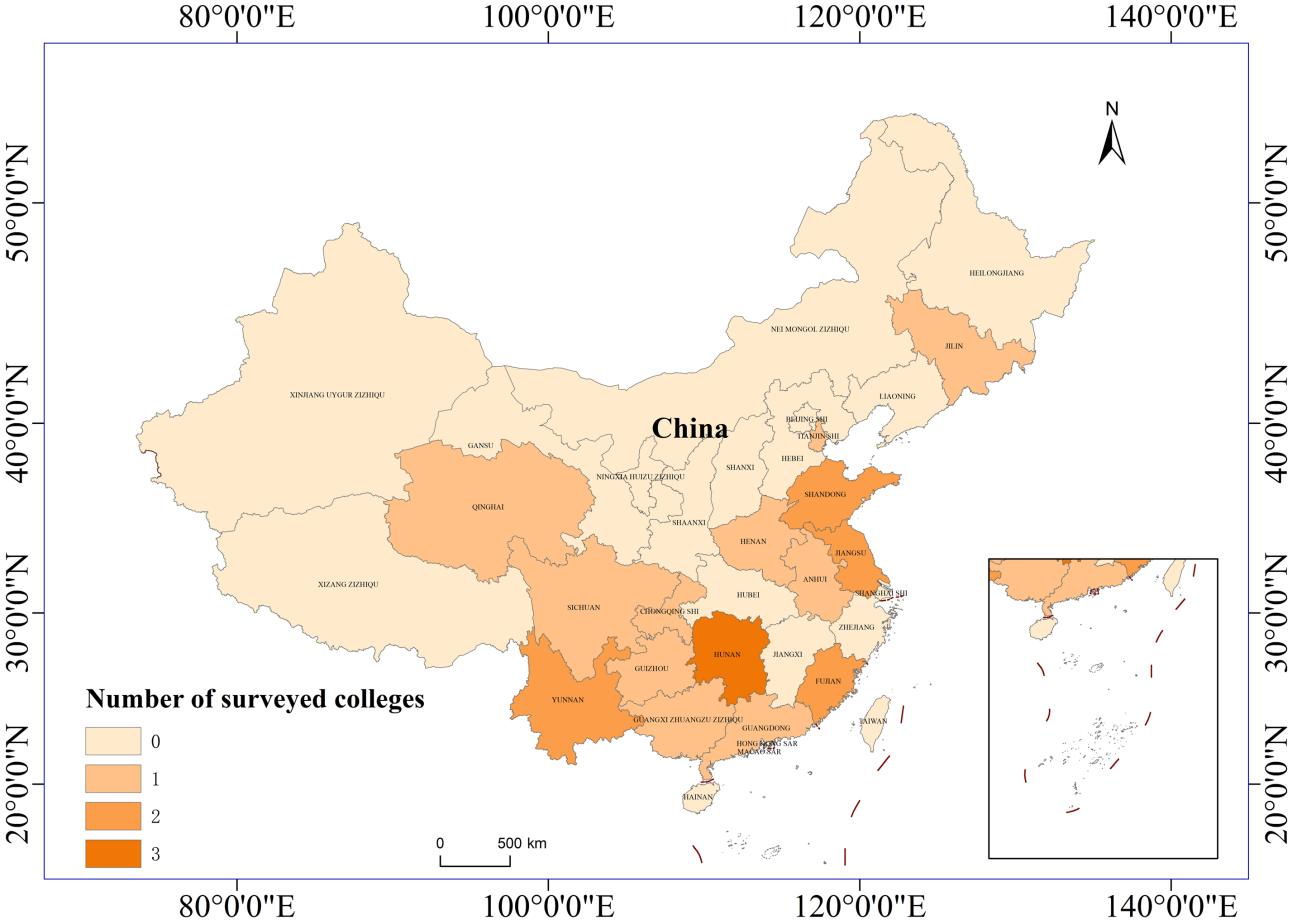
Infographics of the studied institutions.

A multistage selection process was used. First, we selected 13 provinces and two directly administered municipalities across eastern, central, and western China on the basis of economic level and geographical location (Figure 1). (1) In eastern China, the selected provinces were Tianjin, Guangdong, Fujian, Guangxi, Shandong, Jilin, and Jiangsu Provinces. (2) In central China, the selected provinces were Henan, Anhui, and Hunan Provinces. (3) In western China, the selected provinces were Guizhou, Sichuan, Chongqing, Qinghai, and Yunnan Provinces. The classification rules for the eastern, central and western regions were derived from the National Bureau of Statistics of China (22).

To obtain a sufficient sample size, colleges that had more than 30 graduates were included from the chosen provinces and municipalities. Finally, we invited graduate students attending each college since 2016 or later who are currently working at the Centers for Disease Control (CDCs) and medical institutions to participate.

Overall, 20 medical colleges with 1,083 preventive medicine graduate students working at the CDCs and medical institutions were included in this study.

### 2.2 Data collection

Self-designed questionnaires on the Questionnaire Star platform were used to collect the data. The participants completed the questionnaire by scanning QR codes distributed on WeChat.

### 2.3 Ethics statement

The study was approved by the ethics committee of Fujian Health College (approval number: RT2023-01; approval date: May 15, 2023).

### 2.4 Establishing a talent evaluation index system

#### 2.4.1 Indicator explanations and estimation method

The talent evaluation index system consists of four layers—personal qualities (PQs), professional knowledge (PK), professional skills (PSs), and comprehensive competencies (CCs)—and includes specific requirements for each layer. (1) PQs include core values, humanities and psychological qualities, collaboration and innovation and foundational comprehensive abilities. (2) PK includes medical foundations, preventive medicine core modules, psychology and ethics, computer applications and health laws and regulations. (3) PSs encompass a range of specific operational capabilities across multiple areas, including environmental and occupational health testing, food hygiene and nutrition, and child and adolescent health. (4) CCs include information processing, communication and teamwork skills and thematic report writing skills. There are 10, 26, 55, and 8 courses or skills under these four indicators, respectively. A detailed list of the talent evaluation index system is provided in Supplementary Questionnaire 1.

The participants needed to evaluate whether the medical college from which they graduated provided them with enough training in the talent evaluation index system to meet the requirements of their job positions. There were five options to choose from for every course or skill: ⑤ very adequate, ④ adequate, ③ essentially adequate, ② not adequate, and ① extremely inadequate.

#### 2.4.2 Entropy weight method

The entropy weight method, which was proposed by Shannon (23), is a method for measuring the degree of disorder in a system. The greater the numerical variation in an indicator is, the smaller the information entropy value calculated is; this is because indicators with greater variation contain more information. In other words, it measures the relative strength to represent the average intrinsic information. The steps of the model are as follows:

(1) Normalize the original data

The evaluation index matrix of talent quality is set as $x_{\mathrm{ij}}$ , where i = 1, 2, …, m and m is the number of medical vocational colleges; and where j = 1, 2, …, n and n is the number of evaluation indicators:


$$X={\left(x_{\mathrm{ij}}\right)}_{m\times n}(i=1,2,\ldots ,20;j=1,2,\ldots ,20)$$ (1)

To obtain the standardized evaluation matrix, the extreme value standardization method is used to transform the original index.

Positive indicators


$$x_{\mathrm{ij}}^{'}=\frac{x_{\mathrm{ij}}-\min(x_{1j,\ldots ,}x_{\mathrm{mj}})}{\max(x_{1j,\ldots ,}x_{\mathrm{mj}})-\min(x_{1j,\ldots ,}x_{\mathrm{mj}})}$$ (2)

Negative indicators


$$x_{\mathrm{ij}}^{'}=\frac{\max(x_{1j,\ldots ,}x_{\mathrm{mj}})-x_{\mathrm{ij}}}{\max(x_{1j,\ldots ,}x_{\mathrm{mj}})-\min(x_{1j,\ldots ,}x_{\mathrm{mj}})}$$ (3)

(2) Estimate the weight of indicator j:

$$p_{\mathrm{ij}}=\frac{x_{\mathrm{ij}}^{'}}{\sum_{i=1}^{20}x_{\mathrm{ij}}^{'}}$$ (4)

$$H_{j}=-\frac{\sum_{i=1}^{20}p_{\mathrm{ij}}\ln p_{\mathrm{ij}}}{\ln20}$$ (5)

$$w_{j}=\frac{1-H_{j}}{\sum_{j=1}^{20}1-H_{j}}$$ (6)

where $H_{j}$ is the information entropy. $w_{j}$ is the weight of indicator *j* and $w_{j}$ ∈ [0,1].

(3) Estimate the comprehensive score for the preventive medicine talent quality of a given medical vocational college:

$$S_{i}=\sum_{j=1}^{20}w_{j}p_{\mathrm{ij}}$$ (7)

### 2.5 Data analysis

After the score data for courses or skills were collected, the average score for courses or skills was used as the indicator score. On the basis of the entropy weight method, we obtained the weights of the indicators. The weights of the layers, which include the PQ, PK, PS, and CC layers, were obtained by summing the weights of the indicators. In terms of the comprehensive score, we calculated the result in two ways:

(1) At the layer level, the comprehensive scores for PQs, PK, PSs, and CCs were calculated by Equations 1−7 for different colleges.
(2) The comprehensive scores of talent quality for different colleges were equal to the sum of the comprehensive scores for PQs, PK, PSs, and CCs.

The mean and standard deviation were used to determine descriptive statistics. For different regions of China, we took the average score of the corresponding colleges as the final score.

All analyses were performed via IBM SPSS Statistics for Windows, version 26 (IBM Corp., Armonk, N.Y., USA).

### 2.6 Validity and reliability of the questionnaires

A rigorous development and validation process was used to ensure the validity of the questionnaire. The development and validation process for this questionnaire strictly adhered to the guidelines of the National Health Vocational Education Teaching Steering Committee. We established a dedicated expert working group comprising 21 experts, including professors and specialists from medical vocational colleges offering preventive medicine programs, to ensure the authority and professionalism of the measurement tool. In addition, eight distinguished industry experts at the national, provincial, and municipal levels of the CDC system were invited to serve as senior advisors, contributing valuable practical perspectives from the field. Collectively, this multidisciplinary panel of 29 experts was responsible for the validation of both the research design and the questionnaire instrument. Ambiguous or less relevant items were revised or eliminated through repeated discussions and consensus building, ensuring that the final instrument fully covered the core dimensions of professional competencies. Additionally, prior to the cross-sectional survey, we conducted a pilot study with a small sample of the target population (*n* = 60) to further refine the wording and structure of the questionnaire, confirming that the items were clearly understood and relevant to actual circumstances.

A Cronbach’s *α* reliability analysis of the questionnaire revealed an overall Cronbach’s α of 0.994, and the Cronbach’s α coefficients of the four layers were 0.994, 0.983, 0.994, and 0.989, respectively. All the coefficients were above 0.90. The results indicated that the reliability of the questionnaire was acceptable.

## 3 Results

### 3.1 Evaluation index system for talent quality

Table 1 shows the weights of the indicators and layers calculated using the entropy weight method. According to the calculation results, regardless of the minimal contribution of personal qualities (0.1430), the weight of professional skills was 0.4118, making it the most important factor affecting the comprehensive score for preventive medicine talent quality among the 20 medical vocational colleges. Among the indicators, health laws and regulations accounted for the greatest weight (0.0712), followed by data analysis and statistics (0.0691) and computer applications (0.0686). These results illustrate the important role of professional skills and professional knowledge in this evaluation framework.

**Table 1:** Evaluation index system for preventive medicine talent quality.

Layer	Indicator	Attribute	Weight
Personal qualities (0.1430)	Foundational Comprehensive Abilities	+	0.0368
	Core Values	+	0.0354
	Humanities and Psychological Qualities	+	0.0354
	Collaboration and Innovation	+	0.0353
Professional knowledge (0.2993)	Health Laws and Regulations	+	0.0712
	Computer Applications	+	0.0686
	Psychology and Ethics	+	0.0618
	Core Preventive Medicine	+	0.0593
	Medical Foundations	+	0.0383
Professional skills (0.4118)	Data Analysis and Statistics	+	0.0691
	Child and Adolescent Health	+	0.0622
	Clinical Skills	+	0.0544
	Environmental and Occupational Health Testing	+	0.0504
	Epidemiology	+	0.0467
	Food Hygiene and Nutrition	+	0.0446
	Health Education	+	0.0436
	Public Health Operational Skills	+	0.0408
Comprehensive competencies (0.1459)	Thematic Report Writing Skills	+	0.0590
	Communication and Teamwork Skills	+	0.0459
	Information Processing	+	0.0410

### 3.2 Evaluation results regarding talent quality

On the basis of the evaluation index system established in this paper, the PQ, PK, PS, and CC scores of 20 medical vocational schools were calculated. On the basis of the weight of each indicator, we calculated the talent quality of each medical vocational school. The results are shown in Table 2. The average score for talent quality was 88.00, with the highest score of 95.47 achieved by College A4 in Shandong Province.

**Table 2:** Evaluation results regarding talent quality in different medical vocational colleges.

School	Region	Province	Talent quality	Rank	Personal qualities	Rank	Professional knowledge	Rank	Professional skills	Rank	Comprehensive competencies	Rank
A4	Eastern	Shandong	95.47	1	13.58	1	28.80	1	39.27	1	13.82	1
A12	Eastern	Jilin	91.77	2	12.87	10	27.73	3	37.93	2	13.24	3
A13	Central	Hunan	91.76	3	13.02	6	27.71	5	37.84	3	13.19	5
A7	Eastern	Jiangsu	90.91	4	12.84	11	27.63	6	37.36	4	13.07	8
A8	Eastern	Fujian	90.88	5	12.93	8	27.90	2	37.10	5	12.95	11
A18	Western	Qinghai	90.84	6	13.09	5	27.58	7	37.08	6	13.09	7
A9	Central	Henan	90.56	7	13.38	3	27.49	8	36.44	9	13.25	2
A5	Eastern	Shandong	90.55	8	13.24	4	27.73	4	36.38	10	13.20	4
A6	Eastern	Jiangsu	89.83	9	12.71	13	27.47	9	36.77	7	12.89	12
A19	Western	Yunnan	89.67	10	13.42	2	27.03	11	36.05	11	13.17	6
A14	Central	Hunan	89.09	11	13.00	7	26.46	15	36.63	8	13.00	9
A11	Central	Hunan	88.75	12	12.77	12	27.03	10	35.95	12	13.00	10
A15	Western	Guizhou	87.82	13	12.54	14	26.93	12	35.88	13	12.48	14
A10	Central	Anhui	86.82	14	12.87	9	26.66	13	34.75	16	12.53	13
A3	Eastern	Fujian	86.12	15	12.44	15	26.52	14	34.92	15	12.25	15
A1	Eastern	Tianjin	85.47	16	12.09	16	26.44	16	35.01	14	11.93	17
A17	Western	Chongqing	83.57	17	12.03	17	25.76	17	33.77	18	12.02	16
A2	Eastern	Guangdong	81.04	18	10.51	20	25.58	18	34.18	17	10.77	20
A16	Western	Sichuan	79.65	19	11.93	18	24.81	20	31.51	19	11.40	19
A20	Western	Yunnan	79.39	20	11.59	19	25.26	19	31.00	20	11.55	18
Mean			88.00		12.64		26.93		35.79		12.64	
SD*			4.31		0.73		1.00		2.05		0.76	

SD: Standard deviation.

With respect to the indicators, A4 ranked first not only in terms of talent quality but also in terms of every indicator. A12 in Jilin Province and A13 in Hunan Province followed closely behind. The average scores for the four dimensions were 12.64, 26.93, 35.79, and 12.64 points. Among the four layers, PSs showed the greatest variation (SD = 2.05), followed by PK (SD = 1.00). In terms of the entropy weighting method, this explains why the PS layer had the highest weight among all the layers.

### 3.3 Regional score comparison

Table 3 shows the average score and standard deviation of the talent quality scores for each region. In terms of talent quality, the central region ranked first (89.4), followed closely by the eastern region (89.11), and the western region ranked third (85.16). In terms of the four indicators, the central region ranked first in terms of PQs and CCs, while the eastern region ranked first in terms of PK and PSs.

**Table 3:** The average score and standard deviation of the talent quality scores for each region.

Region	Talent quality	Personal qualities	Professional knowledge	Professional skills	Comprehensive competencies
Central China	89.4(±1.88)	13.01(±0.23)	27.07(±0.53)	36.32(±1.12)	12.99(±0.28)
Eastern China	89.11(±4.24)	12.58(±0.89)	27.31(±0.96)	36.55(±1.62)	12.68(±0.9)
Western China	85.16(±5.02)	12.43(±0.71)	26.23(±1.11)	34.21(±2.54)	12.28(±0.76)

*Data in the table are expressed as the mean (±SD).

**Figure 2:**
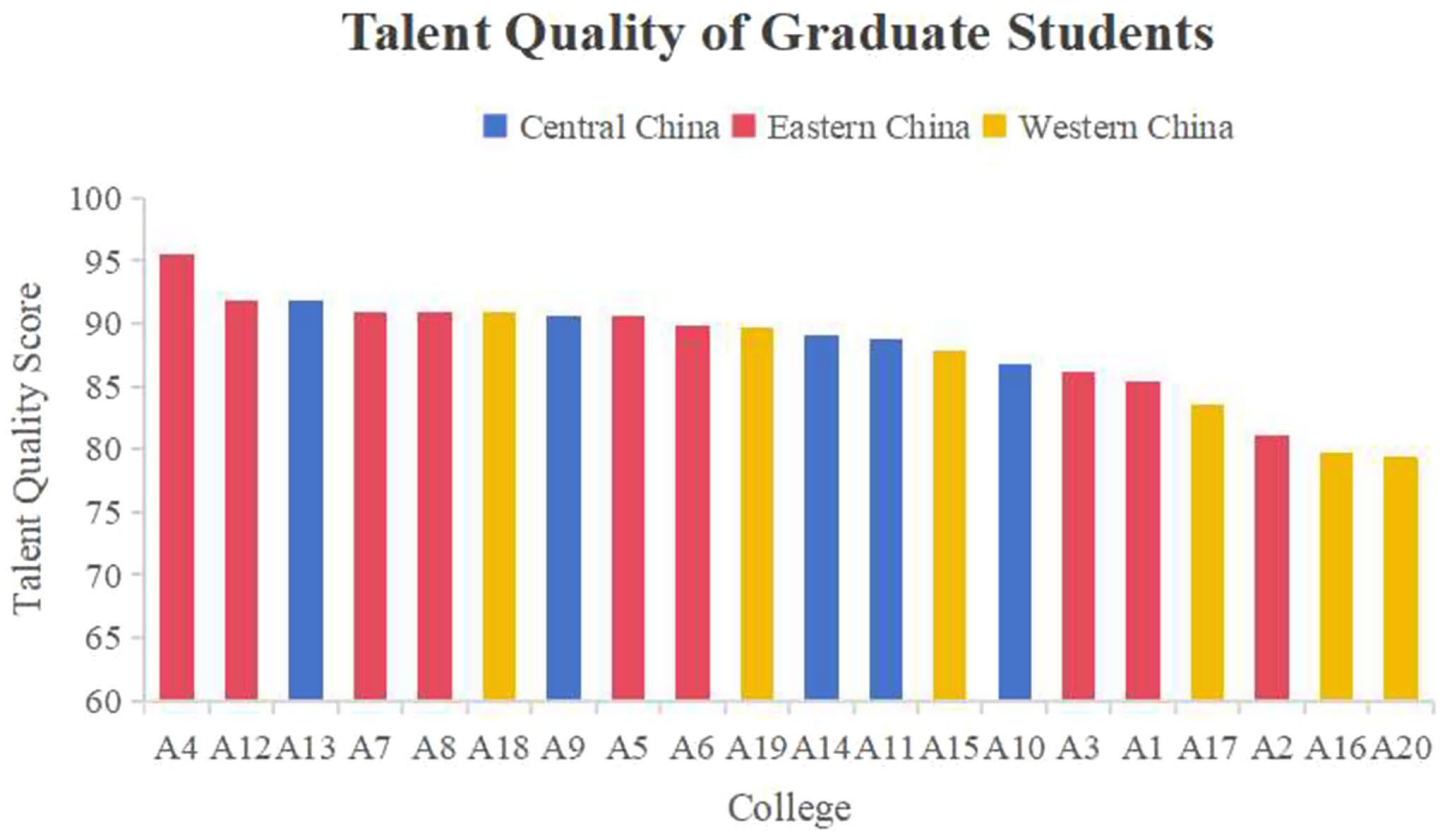
Talent quality score rankings of different colleges.

The talent quality score rankings of the different colleges are shown in Figure 2. The colleges ranked 1st, 2nd, 4th, and 5th were all in eastern China, and the first-place score was significantly higher than the second-place score, but College A2 from eastern China had a very low score. The central region ranked third and occupied the middle part of the ranking. The schools ranked in the middle were mainly in central China. Only two schools in western China ranked in the top 10, while 3 of the bottom four schools were in western China.

## 4 Discussion

### 4.1 Implications

Overall, the average score for talent quality in China was 88, which is considered acceptable. The school that ranked first had a score exceeding 95 points. Specifically, the overall score of College A4 not only greatly exceeded that of the other schools but also ranked first in all layers. The reason is that the preventive medicine program at College A4 in Shandong Province adopts a “1 + 1 + 1” segmented training approach. In the first year, students study medical and professional foundation courses on campus. In the second year, they receive clinical and professional course instruction and participate in internships at the LuNan Public Health College. In the third year, they intern at various CDC levels. Compared with other schools, the highlight of this program at this school is the on-campus and off-campus internships and practical training that begin in the second year. This unique training method has created a new situation of integration between industry and education, enabling students to adapt to their future jobs in advance. This work experience enhances students’ PK and PSs. Working with colleagues and communicating with leaders also improve their CCs. In addition, the employment rate of College A4 graduates has consistently remained above 90%, with a job placement rate of approximately 95% in related fields, and the rate of employer satisfaction with graduates is maintained at 100% satisfaction rate. These findings also reflect the high quality of talent cultivation at College A4 and its significant social influence (24).

In terms of different regions in China, there is little difference in talent quality scores between the central and eastern regions, with the scores of both far exceeding those of the western region. Previous studies have shown that eastern China has developed faster than central China has (25, 26). Furthermore, there is a talent saturation phenomenon in more developed cities in eastern China (27). However, the talent quality score of central China exceeded that of eastern China in our study. This may be related to the fact that, although A2 University is located in eastern China, its host city, Zhaoqing, is geographically close to western China and is relatively less economically developed. YANG Wei et al. reported that Zhaoqing has relatively lagging economic development in the Greater Bay Area and is a “depressed area” in this region with high-quality development. Compared with other developed cities, Zhaoqing lags far behind and experiences imbalanced development (28). This is consistent with the rank in our results. If College A2 was excluded, the talent quality score for eastern China reached 90.12, surpassing the scores for central and western China. As mentioned earlier, four of the top five colleges in terms of talent quality were located in eastern China. These findings are consistent with China’s overall regional development trend (29). First, economic factors are linked to disparities in talent quality. China’s strategies and measures have resulted in rapid economic development in the eastern region (26). Moreover, the eastern region has geographical advantages and abundant resources, as well as a high level of financial technology innovation (30). Second, investment in health care resources affects the development of medical colleges. Chen et al. reported that health funding, human resources, and physical resources tend to be concentrated in wealthier regions, with an overall increasing trend from western to eastern China (25, 31). Third, the quality of talent may be related to disparities in the development of medical and health care talent in the region. Lei Zhang et al. reported significant disparities in the quantity of medical and health talent between the eastern and western regions of China (32). These findings are consistent with the fact that the regions with the highest health care worker density index are in eastern provinces, whereas those with the lowest are in western provinces such as Tibet, Qinghai, and Xinjiang. Furthermore, this gap has continued to widen over the past decade (33–35). The development of health care talent provides not only talent for regional medical and health care systems but also talent for school preventive medicine programs. These regional differences are consistent with the trends identified in our study.

On the basis of the results of the comprehensive evaluation analysis, each medical college has areas for improvement in terms of PQs, PK, PSs, and CCs. The reason may be that many medical colleges started public health education relatively late, and the level of emphasis on public health is far lower than that on clinical medicine, nursing, and other medical specialties. Some colleges have relatively little investment in resources for public health teaching, research, internships, and other aspects, and highly skilled professionals in the fields of disease prevention and control, infectious disease prevention and control, and emergency management for sudden incidents are lacking (36).

College A12 in Jilin Province and College A7 in Jiangsu Province ranked second and fourth, respectively, in terms of talent quality, but their PQ rankings were tenth and eleventh, respectively, indicating that PQs need urgent improvement at these colleges. Jane Adam et al. reported that assessing noncognitive personal qualities among medical school applicants may serve as a useful supplement to admissions selection decisions (37). In the process of transitioning from being a student to being a medical professional, students often encounter problems such as reduced perceptions of self-competence and unreadiness for practice (38–40), overwhelming feelings of inadequacy (39) and reduced self-confidence (40–42). This reality indicates that some gaps may still exist between students’ PQs and job requirements.

The PK scores of College A16 in Sichuan Province and College A20 in Yunnan Province were low, and their PK sections require optimization. In 2004, the Association of Schools of Public Health (ASPH) established a core competencies framework for public health programs, covering five disciplinary areas: environmental health, epidemiology, biostatistics, social and behavioral sciences, and health policy and management (43). Furthermore, the Council on Education for Public Health (CEPH) developed the Accreditation Criteria for Schools of Public Health & Public Health Programs, which stipulate specific requirements for the basic knowledge and skills that public health graduates at the bachelor’s, master’s, and doctoral levels should possess (44). However, no universally agreed-upon core competency model is available in China at present, and domestic research on the core competency indicator system for public health professionals remains relatively scarce.

Our research revealed that the standard deviation of PS was the greatest in the four layers, indicating differences in the degree of PS among schools. Practical skill instruction is always ignored in many higher education institutions in China when talent is cultivated in preventive medicine (45). This is primarily attributed to the fact that the lack of prescribing authority has resulted in talent having few opportunities for professional training and learning (46). According to a survey on the integration of medical and preventive medicine at the grassroots level in 2020, the main issues in this area include insufficient clinical knowledge among public health service personnel (53.2%) (47). The professional skills of preventive medicine students clearly need to be improved. Students also have a high demand for professional knowledge and practical skills. Research has shown that 92.8% of students aspire to improve their knowledge and practical skills in preventive medicine (36). Jinjuan et al. reported that the ratio of experimental class hours to theoretical class hours in 10 preventive medicine programs was less than 1, which is significantly lower than that of clinical medicine programs (48).

The CC layer included some generic skills. College A2 in Guangdong Province had a very low CC score and was the only college with a CC score less than 11 points. The labor market is placing increasingly high demands on students’ generic skills (49, 50). Higher education institutions have explored how to integrate these skills into higher education lessons, which involves integrating creating self-directed study components and educational programs that prioritize the development of essential generic skills (51, 52). However, employers remain dissatisfied with graduates, primarily because of the overly narrow scope of skills and attributes that they possess. Challenges and obstacles continue to exist between employers and individuals in charge of higher education institution policies. These difficulties are especially pronounced because of differences in mentalities, anticipations, and areas of focus (53).

### 4.2 Suggestions

First, medical colleges can develop competency frameworks on the basis of occupational categories and occupational standards. In particular, they need to focus on the relevance and practicality of students’ future work, promote creativity, cultivate creative thinking, foster skills that align with job requirements, emphasize the cultivation of practical skills, and enhance students’ ability to solve practical problems and handle onsite situations (54). For example, medical colleges can offer practical teaching courses such as “Comprehensive Public Health Skills,” “Public Health Service Learning and Practice,” “Community Health Service Management,” “Safety Risk Theory and Emergency Skills Practice,” “Emergency Management of Public Health Emergencies,” and “Basic Theory and Practice of Epidemiology”. To fulfill the requirements of PQs, colleges can resolve this issue in two ways: (1) fostering enthusiasm for acquiring new competencies and advancing professional growth (55, 56) and (2) strengthening motivation by connecting students’ professional roles to spiritual benefits (57). Moreover, medical colleges need to refer to employers’ thinking and develop some generic skills courses, such as teamwork skills, communication skills at work, and work form processing. With respect to communication and teamwork skills, the interprofessional Peer Teacher Training (PTT) program developed by Burgess et al. has a well-validated framework that can be adapted to preventive medicine education (58). On the basis of this model, we recommend integrating structured communication frameworks (e.g., ISBAR: Introduction, Situation, Background, Assessment, and Recommendations) into preventive medicine curricula; organizing interprofessional small-group activities to foster mutual understanding; and adopting a flipped learning approach that reserves face-to-face time for active practice with immediate feedback. With respect to information processing, educators can integrate the AI-enhanced trade simulation developed by Patil et al. into preventive medicine curricula, providing students with structured opportunities to practice data collection, analysis, and interpretation (59). Through guided engagement with such AI tools, students can also be supported in developing professional information acquisition ability, computer skills, literature retrieval ability, and thematic report writing ability.

Second, with reference to the U.S. public health education accreditation system and the core competencies framework, China’s accreditation system for clinical medicine and other related majors, it is recommended to develop a core competency model for public health professionals that embodies Chinese characteristics and meets job requirements. This model should clearly define competency expectations, including PQs, PK, PSs and CCs. A corresponding competency evaluation system is also needed.

Third, it is recommended that public health physicians in all positions be granted the right of prescription, especially for those in public health positions, for which there is much work related to clinical applications, such as infectious disease prevention and control, chronic disease prevention and control, occupational health, health care, and general public health (60). This can motivate medical colleges and students to strive to enhance their PK.

Finally, the objective weighting method employed in this study provides a data-driven foundation for the cross-institutional assessment of talent quality. However, it should be noted that a low entropy value should not be misinterpreted as indicating low practical significance. Rather, it reflects the degree of variation in that indicator across institutions. When using the evaluation results for policymaking or resource allocation, we recommend that administrators integrate the objective evaluation results with clinical and public health priorities to ensure that competencies of high importance are fully considered in final decisions, even if these competencies are well taught across all institutions.

### 4.3 Limitations

This study has several limitations. First, cross-sectional data were used. We cannot observe changes in the talent quality of each school over time. Therefore, we cannot draw reliable causal inferences. In addition, future research should prioritize the collection of longitudinal follow-up data spanning multiple time points and incorporate a broader range of influencing factors to better elucidate the temporal evolution of talent quality and provide a basis for formulating more definitive policy recommendations.

Our data collection was not sufficiently comprehensive. Owing to the limited number of graduates with degrees in preventive medicine in some regions, which did not meet our research requirements, we collected data from only 13 provinces and two directly administered municipalities. In the future, we will consider collecting more data on preventive medicine graduates from medical colleges.

## 5 Conclusion

In contrast to other studies evaluating preventive medicine talent, this study used the entropy weight method to conduct a comprehensive evaluation based on data from graduate students in Chinese medical vocational colleges, eliminating subjective interference. The average talent quality score in China was 88.39 points, which is acceptable. The top-ranked school is located in Shandong Province, and its score exceeded 95 points. The comprehensive evaluation results revealed that the scores are high in the eastern and central regions and low in the western region, which may be related to regional development. We suggest improvements in the PQ, PK, PS, and CC layers. To improve the quality of graduate students, colleges and relevant departments should focus on curricula that align with future work environments, accreditation for public health and preventive medicine, and the prescribing authority of public health physicians.

## Data Availability

These datasets can be obtained from the corresponding author upon reasonable request, pending approval from the National Disease Control and Prevention Administration of China.

## References

[1] Chen X Giles J Yao Y Yip W Meng Q Berkman L . The path to healthy ageing in China: a Peking University-lancet commission. Lancet. (2022) 400:1967–2006. doi: 10.1016/S0140-6736(22)01546-X, 36423650 PMC9801271

[2] Chinese Ministry of Health. Medium- and long-term plan for the development of medical and health talent (2011–2020). Available online at: https://www.nhc.gov.cn/wjw/gfxwj/201104/b4a3dffd6f574945b6e16fd924d173e1.shtml (Accessed September 15, 2025).

[3] National Health Commission of the People's Republic of China. 2023 China health yearbook. Available online at: https://www.nhc.gov.cn/mohwsbwstjxxzx/tjtjnj/202501/8193a8edda0f49df80eb5a8ef5e2547c.shtml (Accessed September 15, 2025).

[4] Rounds K. Public health social work standards and competencies. Available online at: https://www.brynmawr.edu/sites/default/files/migrated-files/Public%20Health%20Social%20Work%20Standards%20and%20Competencies.pdf (Accessed September 15, 2025).

[5] Benavides FG Moya C Segura A de la Puente ML Portaa M Amela C . Las competencias profesionales en Salud Pública [Professional competencies in public health]. Gac Sanit. (2006) 20:239–43. doi: 10.1157/13088856 16756863

[6] Leethongdissakul S Chada W Pengpid S Chualinfa S. An exploratory factor analysis of core competencies of public health professionals at primary care service level in northeastern Thailand. SAGE Open Med. (2020) 8:2050312120940531. doi: 10.1177/2050312120940531, 33062274 PMC7533937

[7] Nelson-Hurwitz DC Tagorda M Kehl L Buchthal OV Braun KL. Developing an undergraduate public health introductory core course series. Front Public Health. (2018) 6:155. doi: 10.3389/fpubh.2018.00155, 29892596 PMC5985697

[8] Brînzac MG Verschuuren M Leighton L Otok R. Public health competencies: what does the next generation of professionals deem important? Eur J Pub Health. (2025) 35:ii11–6. doi: 10.1093/eurpub/ckae201, 40130373 PMC11933798

[9] Warren R Young L Carlisle K Heslop I Glass B. Identifying public health competencies for Australian pharmacists: a modified Delphi study. Aust N Z J Public Health. (2025) 49:100210. doi: 10.1016/j.anzjph.2024.100210, 39818029

[10] Tang Y Wang C Zhang J Li W. Research on the curriculum system for training public health talents based on post competency. Course Educ Res. (2018) 21:10–1.

[11] Zhang L Pei H Niu J Li X Zhang X Zhang K . Construction of innovative practical skill curriculum system for undergraduate public health core majors under the guidance of "healthy China". College Lab Sci Technol. (2019):29–31.

[12] Torok MR White AE Thompson S Pugh S Garman K Anand M . Competencies for environmental health professionals who detect, investigate, and respond to foodborne illness outbreaks. J Environ Health. (2022) 85:24–33.37206159

[13] Hughes R. Competencies for effective public health nutrition practice: a developing consensus. Public Health Nutr. (2004) 7:683–91. doi: 10.1079/PHN2003574, 15251059

[14] Van der Putten MG Vichit-Vadakan N Chuchat A Van Der Putten M Love E. Assessing the required skill mastery in public health competencies in Thailand. Educ Health Change Learn Pract. (2006) 19:233–43. doi: 10.1080/13576280600783844, 16831805

[15] Adewale O Apenteng BA Shah GH Mase WA. Assessing public health workforce informatics competencies: a study of 3 district health departments in Georgia. J Public Health Manag Pract. (2022) 28:E533–41. doi: 10.1097/PHH.0000000000001393, 34081672

[16] Ministry of Education of the People's Republic of China. Notice of the Ministry of Education on Issuing the “Administrative Measures for the Establishment of Higher Vocational Education (Specialty) Programs in Regular Higher Education Institutions” and the “Directory of Higher Vocational Education (Specialty) Programs in Regular Higher Education Institutions (2015)”. Available online at: http://www.moe.gov.cn/srcsite/A07/moe_953/201511/t20151105_217877.html (Accessed March 26, 2026).

[17] Ministry of Education of the People's Republic of China. Notice of the Ministry of Education on Announcing the Recordation and Approval Results of Higher Vocational Education (Specialty) Program Establishments in Regular Higher Education Institutions for 2016. Available online at: http://www.moe.gov.cn/srcsite/A07/moe_953/201604/t20160401_236222.html (Accessed March 26, 2026).

[18] Ministry of Education of the People's Republic of China. Notice of the Ministry of Education on Announcing the Recordation and Approval Results of Higher Vocational Education (Specialty) Program Establishments in Regular Higher Education Institutions for 2017. Available online at: http://www.moe.gov.cn/srcsite/A07/moe_953/201702/t20170208_295895.html (Accessed March 26, 2026).

[19] Ministry of Education of the People's Republic of China. Notice of the Ministry of Education on Announcing the Recordation and Approval Results of Higher Vocational Education (Specialty) Program Establishments in Regular Higher Education Institutions for 2018. Available online at: http://www.moe.gov.cn/srcsite/A07/moe_953/201802/t20180201_326306.html (Accessed March 26, 2026).

[20] Ministry of Education of the People's Republic of China. Notice of the Ministry of Education on Announcing the Recordation and Approval Results of Higher Vocational Education (Specialty) Program Establishments in Regular Higher Education Institutions for 2019. Available online at: http://www.moe.gov.cn/srcsite/A07/moe_953/201901/t20190121_367541.html (Accessed March 26, 2026).

[21] Yang ZY Zhou M Zhu L Li N. Investigation and analysis of construction of preventive medicine specialty in medical higher vocational and technical education. Vocat Techn Educ. (2020) 41:36–40.

[22] Division on Eastern, Central, Western and Northeastern Regions. National bureau of statistics. Available online at: https://www.stats.gov.cn/english/PressRelease/201911/t20191101_1706472.html (Accessed September 15, 2025).

[23] Shannon CE. A mathematical theory of communication. Bell Syst Tech J. (1948) 27:379–423. doi: 10.1002/j.1538-7305.1948.tb01338.x

[24] Shandong Medical College. Introduction to 2024 preventive medicine program of Shandong Medical College. Available online at: https://zsbgs.sdmc.edu.cn/info/1003/1630.htm (Accessed September 15, 2025).

[25] Guo Q Luo K Hu R. The spatial correlations of health resource agglomeration capacities and their influencing factors: evidence from China. Int J Environ Res Public Health. (2020) 17:8705. doi: 10.3390/ijerph17228705, 33238597 PMC7700579

[26] Shen H Teng F Song J. Evaluation of spatial balance of China’s regional development. Sustainability. (2018) 10:3314. doi: 10.3390/su10093314

[27] Ren T Lyu J Yu C Li L. Rethinking public health education and public health workforce development in China. Chinese J Prevent Med. (2020) 54:457–64. doi: 10.3760/cma.j.cn112150-20200330-00473, 32234129

[28] Yang W. Study on the problems and countermeasures in promoting the development of the city of backwardness in Guangdong Hong Kong-Macao Greater Bay Area ——taking Zhaoqing as an example. Bay Area Econ Res. (2025) 1:25–9.

[29] Lv C Bian B Lee C Lee C-C He Z. Regional gap and the trend of green finance development in China. Energy Econ. (2021) 102:105476. doi: 10.1016/j.eneco.2021.105476

[30] Zhou G Zhu J Luo S. The impact of fintech innovation on green growth in China: mediating effect of green finance. Ecol Econ. (2022) 193:107308. doi: 10.1016/j.ecolecon.2021.107308

[31] Chen J Lin Z Li LA Li J Wang Y Pan Y . Ten years of China’s new healthcare reform: a longitudinal study on changes in health resources. BMC Public Health. (2021) 21:2272. doi: 10.1186/s12889-021-12248-9, 34903184 PMC8670033

[32] Zhang L Tang J Zhou Q Song Y Zhang D. Spatial-temporal distribution and evolution of medical and health talents in China. BMC Public Health. (2025) 25:124. doi: 10.1186/s12889-025-21324-3, 39794726 PMC11720572

[33] Bai Q Ke X Huang L Liu L Xue D Bian Y. Finding flaws in the spatial distribution of health workforce and its influential factors: an empirical analysis based on Chinese provincial panel data, 2010–2019. Front Public Health. (2022) 10:953695. doi: 10.3389/fpubh.2022.953695, 36589992 PMC9794860

[34] Lu H Hou L Zhou W Shen L Jin S Wang M . Trends, composition and distribution of nurse workforce in China: a secondary analysis of national data from 2003 to 2018. BMJ Open. (2021) 11:e47348. doi: 10.1136/bmjopen-2020-047348, 34706946 PMC8552175

[35] Zhu B Hsieh C Zhang Y. Incorporating spatial statistics into examining equity in health workforce distribution: an empirical analysis in the Chinese context. Int J Environ Res Public Health. (2018) 15:1309. doi: 10.3390/ijerph15071309, 29932139 PMC6068954

[36] Shao J Hu B Ren X Zhou L Li Y Han Y . Exploration on training strategies for preventive medicine personnel based on post competency in the perspective of public health in the new era. Health Vocat Educ. (2024) 42:142–5. doi: 10.20037/j.issn.1671-1246.2024.22.38

[37] Adam J Bore M McKendree J Munro D Powis D. Can personal qualities of medical students predict in-course examination success and professional behaviour? An exploratory prospective cohort study. BMC Med Educ. (2012) 12:69. doi: 10.1186/1472-6920-12-69, 22873571 PMC3473297

[38] Opoku EN Khuabi LJ Van Niekerk L. Exploring the factors that affect the transition from student to health professional: an integrative review. BMC Med Educ. (2021) 21:558. doi: 10.1186/s12909-021-02978-0, 34727905 PMC8561904

[39] Seah CH Mackenzie L Gamble J. Transition of graduates of the master of occupational therapy to practice. Aust Occup Ther J. (2011) 58:103–10. doi: 10.1111/j.1440-1630.2010.00899.x, 21418233

[40] Uys ME Buchanan H Van Niekerk L. Strategies occupational therapists employ to facilitate work-related transitions for persons with hand injuries: a study protocol for a scoping review. BMJ Open. (2019) 9:e27402. doi: 10.1136/bmjopen-2018-027402, 30975685 PMC6500360

[41] Doherty G Stagnitti K Schoo AMM. From student to therapist: follow up of a first cohort of bachelor of occupational therapy students. Aust Occup Ther J. (2009) 56:341–9. doi: 10.1111/j.1440-1630.2008.00751.x, 20854540

[42] Maguire M Delahunt B. Doing a thematic analysis: a practical, step-by-step guide for learning and teaching scholars. All Ireland J High Educ. 9:3351. doi: 10.62707/aishej.v9i3.335

[43] Calhoun JG Ramiah K Weist EM Shortell SM. Development of a core competency model for the master of public health degree. Am J Public Health. (2008) 98:1598–607. doi: 10.2105/AJPH.2007.117978, 18633093 PMC2509588

[44] Council on Education for Public Health. Accreditation criteria for schools of public health & public health programs. Available online at: https://media.ceph.org/documents/2024.Criteria.pdf (Accessed September 15, 2025).

[45] Li X Lv L Li J Yang M Zou K. Research on public health practitioners prescription rights from the perspective ofmanagers of grassroots medical institution in Chengdu City. Med Soc. (2023) 36:74–8. doi: 10.13723/j.yxysh.2023.07.014

[46] Lv L Li X Li J Yang M Zou K. Investigating prescription right of public health physicians from the perspective of primary public health physicians based on grounded theory. Modern Prevent Med. (2023) 50:874–878+939. doi: 10.20043/j.cnki.MPM.202207331

[47] Qu W Chen H Zheng Q Wang M Liu Y Guo Y. Construction and development of grass - roots public health talents from the perspective of medical prevention integration. Chinese Health Serv Manag. (2021) 38:839–843, 846.

[48] Li J Jin H Zhang Y Wu J Yue J Pei L . Thoughts on the talent training model for the preventive medicine major in TCM colleges and universities. Health Vocat Educ. (2015) 33:15–6.

[49] Sarkar M Gibson S Karim N Rhys-Jones D Ilic D. Exploring the use of self-assessment to facilitate health students' generic skills development. J Teach Learn Grad Employ. (2021) 12:65–81. doi: 10.21153/jtlge2021vol12no2art976

[50] Siqueira MAM Torsani MB Gameiro GR Chinelatto LA Mikahil BC Tempski PZ . Medical students’ participation in the volunteering program during the covid-19 pandemic: a qualitative study about motivation and the development of new competencies. BMC Med Educ. (2022) 22:111. doi: 10.1186/s12909-022-03147-7, 35183158 PMC8857627

[51] Jackson D. Self-assessment of employability skill outcomes among undergraduates and alignment with academic ratings. Assess Eval High Educ. (2013) 39:53–72. doi: 10.1080/02602938.2013.792107

[52] Sarkar M Overton T Thompson C Rayner G. Undergraduate science students' perceptions of employability: efficacy of an intervention. Int J Innov Sci Math Educ. (2017) 25:21–37.

[53] Lowden K Hall S Elliot D Lewin J. Employers' Perceptions of the Employability Skills of New Graduates. Project Report. London, UK: Edge Foundation.

[54] Wang H Sun M Hou H Pang X Nie L. Systematic design and pilot initiation of the standardized training system for public health physicians in Beijing. Occup Health. (2015) 31:991–4. doi: 10.13329/j.cnki.zyyjk.2015.0396

[55] Clare J Loon AV. Best practice principles for the transition from student to registered nurse. Collegian. (2003) 10:25–31. doi: 10.1016/S1322-7696(08)60073-615470986

[56] Labrague LJ McEnroe-Pettite D Leocadio MC. Transition experiences of newly graduated Filipino nurses in a resource-scarce rural health care setting: a qualitative study. Nurs Forum. (2019) 54:298–306. doi: 10.1111/nuf.12330, 30775787

[57] Al Awaisi H Cooke H Pryjmachuk S. The experiences of newly graduated nurses during their first year of practice in the Sultanate of Oman - a case study. Int J Nurs Stud. (2015) 52:1723–34. doi: 10.1016/j.ijnurstu.2015.06.009, 26164747

[58] Burgess A Roberts C van Diggele C Mellis C. Peer teacher training (PTT) program for health professional students: interprofessional and flipped learning. BMC Med Educ. (2017) 17:239. doi: 10.1186/s12909-017-1037-6, 29202736 PMC5715628

[59] Patil U Terada M Nelson-Hurwitz DC. Global Health trade summit: an AI-enhanced simulation of international trade and global health for undergraduate public health education. Front Public Health. (2025) 13:1681199. doi: 10.3389/fpubh.2025.1681199, 41323585 PMC12660194

[60] Long C Tang S Feng D Zhou J Fu H Li G . Development and improvement of public health physician system in China. Chin J Public Health. (2019) 35:937–40. doi: 10.11847/zgggws1120363

