# Supplementary material for: Comprehensive evaluation of preventive medicine talent in Chinese medical vocational colleges based on the entropy weight method
Source: Front Med (Lausanne). 2026 May 20;13:1749127. doi: 10.3389/fmed.2026.1749127 (PMC13229703; doi:10.3389/fmed.2026.1749127)
Supplement: Supplementary file 1 [file Table_1.DOCX]

**Supplementary Questionnaire 1**

Please evaluate whether the medical college you graduated from provided sufficient training in the following professional modules and skills to meet the requirements of your job position. Five Levels of skills meet the job requirements: ⑤ very adequate ④ adequate ③ essentially adequate ② not adequate ① extremely inadequate

| **Layer** | **Indicator** | **Course or Skill** |
| --- | --- | --- |
| **Personal Qualities** | Core Values | - Moral Character - Ideals and Beliefs - Professionalism |
|  | Humanities and Psychological Qualities | - Life Education - Humanistic Literacy - Psychological Quality |
|  | Collaboration and Innovation | - Innovation Awareness - Teamwork Awareness |
|  | Foundational Comprehensive Abilities | - Comprehensive Judgment Ability - Safety Awareness |
| **Professional Knowledge** | Medical Foundations | - Human Anatomy - Microbiology and Immunology - Physiology - Pharmacology - Biochemistry - Diagnostics - Internal Medicine - Surgery - Obstetrics and Gynecology - Pediatrics - Emergency Medicine - Infectious Diseases |
|  | Preventive Medicine Core Modules | - Epidemiology - Health Statistics - Occupational Health and Occupational Medicine - Environmental Health - Nutrition and Food Hygiene - Health Education and Promotion - Maternal and Child Health Care and Adolescent Health - Basic Public Health Service Technology - Social Medicine - Comprehensive Skills Training in Preventive Medicine (or similar courses) |
|  | Psychology and Ethics | - Psychology - Medical Ethics |
|  | Computer Applications | - Computer Application-related Courses |
|  | Health Laws and Regulations | - Health Laws and Regulations |
| **Professional Skills** | Environmental and Occupational Health Testing | - Determination of Meteorological Factors - Air and Indoor Air Sampling Methods - Determination of Sulfur Dioxide, Particulate Matter, and Nitrogen Oxides in the Atmosphere - Determination of Formaldehyde Concentration in Air - Collection and Pretreatment of Water Samples - Determination of "Three Nitrogens" and Arsenic in Water - Determination of Oxygen Consumption and Dissolved Oxygen in Water - Determination of Total Iron, Total Hardness, and Chloride in Water - Determination of Available Chlorine in Drinking Water, Residual Chlorine, and Chlorine Demand - Determination of Biomarkers of Exposure to Occupational Hazardous Chemicals in Blood and Urine (e.g., Lead, Benzene, etc.) - Determination of Biomarkers of Effect of Occupational Hazardous Chemicals in Blood and Urine (e.g., Determination of Whole Blood Cholinesterase Activity) - Pneumoconiosis Film Reading - Determination of Free Silica Content in Dust - Determination of Total Dust Concentration and Dust Dispersion - Determination of Physical Hazardous Factors (High Temperature, Noise, Vibration, Radiation, etc.) - Factory Field Testing and Investigation Report Writing |
|  | Food Hygiene and Nutrition | - Determination of Protein Content in Food - Protein Efficiency Ratio - Dietary Survey - Determination of Ascorbic Acid, Total Sugar, and Arsenic in Food - Formulation of Nutritional Recipes - Determination of Nitrite - Determination of Food Coloring Content - Analysis of Food Poisoning Cases - Discussion on Food Hygiene Supervision and Management Cases |
|  | Child and Adolescent Health | - Growth and Development Testing and Evaluation Methods - Investigation and Evaluation of Childhood Obesity - Sanitary Investigation and Methods of Classrooms |
|  | Epidemiology | - Measurement of Disease Frequency - Distribution of Diseases - Cross-sectional Study, Cohort Study, Case-Control Study - Evaluation of Disease Screening Methods - Epidemiological Experimental Methods - Epidemiological Bias and Its Control - Judgment of Disease Causality - Outbreak Investigation - Cross-sectional Survey and Analysis of Chronic Diseases - Analysis and Evaluation of Vaccination Effect |
|  | Public Health Operational Skills | - Disinfection and Sterilization Technology - Simulated Handling of Public Health Emergencies - Donning and Doffing of Personal Protective Equipment - Investigation and Analysis of Environmental Epidemiology Data |
|  | Data Analysis and Statistics | - Basic Operation of Common Statistical Software - Statistical Description of Quantitative and Categorical Data - Estimation of Population Mean and Hypothesis Testing - Common Statistical Test Methods (t-test, Chi-square Test) - Survey Design and Experimental Design - Statistical Tables and Charts |
|  | Health Education | - Production of Health Promotion Materials - Methods of Health Education Promotion and Training |
|  | Clinical Skills | - Physical Examination - Cardiopulmonary Resuscitation - Identification of Common Chronic Diseases - Identification of Common Infectious Diseases - Emergency First Aid |
| **Comprehensive Competencies** | Information Processing | - Professional Information Acquisition Ability - Computer Skills - Literature Retrieval Ability |
|  | Communication and Teamwork Skills | - Teamwork Ability - Organizational Coordination Ability - Communication Skills - Social Mobilization Ability |
|  | Thematic Report Writing Skills | - Thematic Report Writing Ability |
